# GSK3β Interacts With CRMP2 and Notch1 and Controls T-Cell Motility

**DOI:** 10.3389/fimmu.2021.680071

**Published:** 2021-12-17

**Authors:** Mobashar Hussain Urf Turabe Fazil, Praseetha Prasannan, Brandon Han Siang Wong, Amuthavalli Kottaiswamy, Nur Syazwani Binte Mohamed Salim, Siu Kwan Sze, Navin Kumar Verma

**Affiliations:** ^1^ Lee Kong Chian School of Medicine, Nanyang Technological University Singapore, Singapore, Singapore; ^2^ Interdisciplinary Graduate Programme, NTU Institute for Health Technologies (HealthTech NTU), Nanyang Technological University Singapore, Singapore, Singapore; ^3^ School of Biological Sciences, Nanyang Technological University Singapore, Singapore, Singapore

**Keywords:** GSK3β, NOTCH1, T-lymphocytes, LFA-1, T-cell migration

## Abstract

The trafficking of T-cells through peripheral tissues and into afferent lymphatic vessels is essential for immune surveillance and an adaptive immune response. Glycogen synthase kinase 3β (GSK3β) is a serine/threonine kinase and regulates numerous cell/tissue-specific functions, including cell survival, metabolism, and differentiation. Here, we report a crucial involvement of GSK3β in T-cell motility. Inhibition of GSK3β by CHIR-99021 or siRNA-mediated knockdown augmented the migratory behavior of human T-lymphocytes stimulated *via* an engagement of the T-cell integrin LFA-1 with its ligand ICAM-1. Proteomics and protein network analysis revealed ongoing interactions among GSK3β, the surface receptor Notch1 and the cytoskeletal regulator CRMP2. LFA-1 stimulation in T-cells reduced Notch1-dependent GSK3β activity by inducing phosphorylation at Ser9 and its nuclear translocation accompanied by the cleaved Notch1 intracellular domain and decreased GSK3β-CRMP2 association. LFA-1-induced or pharmacologic inhibition of GSK3β in T-cells diminished CRMP2 phosphorylation at Thr514. Although substantial amounts of CRMP2 were localized to the microtubule-organizing center in resting T-cells, this colocalization of CRMP2 was lost following LFA-1 stimulation. Moreover, the migratory advantage conferred by GSK3β inhibition in T-cells by CHIR-99021 was lost when CRMP2 expression was knocked-down by siRNA-induced gene silencing. We therefore conclude that GSK3β controls T-cell motility through interactions with CRMP2 and Notch1, which has important implications in adaptive immunity, T-cell mediated diseases and LFA-1-targeted therapies.

## Introduction

In a healthy human under physiological conditions, T-lymphocytes continuously recirculate between the peripheral lymphoid tissues *via* the blood and lymphatic systems to perform an active immune surveillance as well as mount an adaptive immune response. Dysregulation of T-cell recruitment can result in impaired adaptive immunity, recurrent infections, chronic inflammation, and a diverse range of autoimmune diseases (1).

T-cell trafficking is mediated by an active engagement of cell surface receptors, cytoskeletal remodeling, and a broad array of signal transduction processes, including activation/deactivation of kinases and phosphatases (2). In particular, the T-cell αLβ2 integrin lymphocyte function-associated antigen-1 (LFA-1; CD11a/CD18) binds to its ligand intercellular adhesion molecule-1 (ICAM-1) expressed on the endothelium, and this adhesive interaction is crucial for T-cell migration and effector functions (3). The molecular machinery and the downstream pathways triggered by LFA-1 attachment to the ICAM-1 facilitating T-cell motility remain unclear.

The glycogen synthase kinase 3 (GSK3) is a ubiquitous constitutively active serine/threonine kinase that exists in two isoforms, GSK3α and GSK3β, and targets over hundred proteins to regulate context-specific cellular functions (4). Initially uncovered as a key enzyme involved in glycogen synthesis, GSK3β is now known to regulate cell cycle, development, survival, metabolism, and inflammation in multiple cell types (5–8). However, GSK3β involvement in T-cell motility is yet to be fully understood.

The functional activities of GSK3β are regulated by its post-translational modifications, nuclear localization, and interactions with other proteins. The phosphorylation of a Ser9 residue in the N-terminus of the GSK3β protein (pGSK3β-S9) by other kinases, such as Akt, phosphatidylinositol-3-kinase (PI3K), and protein kinase C (PKC) isoforms, inactivates GSK3β (4). This, in turn, drives dynamic fluxes of primed substrates contributing to the regulation of cell/tissue-specific functions. We have previously reported that LFA-1/ICAM-1 ligation in human peripheral blood lymphocyte (PBL) T-cells promotes Th1 polarization through a GSK3β-dependent pathway (9). Here, we identify GSK3β-interacting proteins and show that GSK3β interacts with the Notch1 and collapsing response mediator protein 2 (CRMP2) and regulates T-cell motility.

## Materials and Methods

### Human T-Cell Isolation and Culture

Human primary PBL T-cells were isolated from healthy volunteers or leukocyte reduction system (LRS) cones obtained from the Health Sciences Authority (HSA) of Singapore using Lymphoprep™ density gradient medium (STEMCELL Technologies) and centrifugation as described previously (10). All experiments involving human peripheral blood or components were approved by the Nanyang Technological University Singapore Institutional Review Board (IRB-2018-05-034 and IRB-2014-09-007). The human T-cell line HuT78 was obtained from the American Type Culture Collection (ATCC, Manassas, VA) and cultured in Gibco™ RPMI 1640 medium supplemented with 10% fetal bovine serum, 1 mM sodium pyruvate and antibiotics (penicillin 100 units/ml, streptomycin 100 μg/ml) at 37°C and 5% CO_2_ as described (11).

### Antibodies and Reagents

Anti-GSK3β, anti-pGSK3β-S9, anti-CRMP2, and anti-rabbit antibodies were from Cell Signaling Technology. Anti-pCRMP2-T514 and anti-pericentrin antibodies were from Abcam. Anti-GM130 was from MBL International. IL-2 and stromal cell-derived factor 1 (SDF-1α) were obtained from PeproTech. The GSK3β inhibitor CHIR-99021 was obtained from STEMCELL Technologies. The γ-secretase inhibitor N-[(3,5-difluorophenyl) acetyl]-L-alanyl-2-phenylglycine-1,1-dimethyl ethyl ester (DAPT) was from Merck Millipore. Recombinant human ICAM-1 (rICAM-1) was procured from Sino Biological. Anti-mouse antibody was from Agilent Technologies. Dimethyl sulfoxide (DMSO), poly-l-lysine (PLL), Phalloidin-Alexa Fluor^®^ 647, CellMask™ (Orange), Hoechst-33342, anti-rabbit and anti-mouse fluorescent secondary antibodies, Gibco™ RPMI 1640 and cell culture supplements were purchased from Thermo Fisher Scientific.

### LFA-1/ICAM-1-Induced T-Cell Migration

We used our well-characterized T-cell migration *in vitro* model, where primary or cultured T-cells were seeded on rICAM-1-coated plates and cells were allowed to migrate as described previously (12). Briefly, 5 µg/ml anti-human IgG (Fc specific) in sterile phosphate buffered saline (PBS, pH 7.2) was coated on 6- or 96-well tissue culture plates or 18 mm coverslips overnight at 4°C. After washing with PBS, 1 µg/ml rICAM-1 was added into the wells of the plates/coverslips and incubated for another 2 h at 37°C. Wells of the plates/coverslips were washed with PBS before seeding T-cells (20×10^3^ cells/well in 96-well plate; 200×10^3^ cells/well in 6-well plate) in an activation medium. The activation medium consisted of cell culture medium with added 5 mM MgCl_2_ and 1.5 mM EGTA.

### Live Cell Imaging of LFA-1/ICAM-1-Stimulated Migrating T-Cells

We used an established live cell imaging protocol to quantify T-cell migration by an automated microscopy (13). Briefly, control or pretreated T-cells were stained with CellMask™ and added on an rICAM-1-coated 96-well flat-bottom plate (2×10^4^ cell per well) and cells were allowed to migrate as described above. Live cell migration was recorded using an automated microscope IN Cell Analyzer 2200 (GE Healthcare) equipped with temperature and environmental controls. Cell tracking and measurements of distance were performed using the Imaris software (Andor-Bitplane, Zurich).

### Real-Time Monitoring of T-Cell Migration in 2D and Through Transwell Membranes

Kinetic monitoring of T-cell migration on rICAM-1-coated 2D surfaces and through transwell membrane towards the chemokine SDF-1α was performed using xCELLigence E-Plate 16 and CIM-Plate 16, respectively, and the Real-Time Cell Analysis (RTCA) instrument (Agilent). The E-Plate 16 plates contain gold microelectrodes embedded in the bottom of each well that can continuously monitor the adhesion and spreading of motile T-cells by automatic measurement of the changes in impedance signals. For T-cell 2D migration assays, bottom surfaces of the E-Plate 16 wells were coated with 1 µg/ml rICAM-1 at 37°C for 2 h. T-cells that have been pre-treated under various experimental conditions, as indicated in the corresponding figure legends, were added in the wells of the rICAM-1-coated E-Plate 16 (2×10^4^ cells/well) in 100 µl activation medium in triplicates. Changes in T-cell migratory phenotypes in 2D, including cell adhesion and spreading, were automatically recorded by impedance measurements using the RTCA system. For transwell migration assays, upper chambers of the CIM-plate 16 plates containing electronically integrated microporous membranes (pore size 8 µm) were coated with 1 µg/ml rICAM-1 at 37°C for 2 h, as describes earlier (14). T-cells that have been pre-treated under various experimental conditions, as indicated in the corresponding figure legends, were loaded in the upper chambers of the CIM-Plate 16 (1×10^5^ cells/well) in 100 µl activation medium in triplicates. Cells were allowed to transmigrate through the membrane toward 100 ng/ml SDF-1α-enriched medium in the lower wells at 37°C. T-cells passing through the pores of the rICAM-1-coated membrane were immediately detected by gold electrodes, covering the lower side of the membrane, and quantified by the RTCA system in terms of impedance changes in real-time. The kinetic data (baseline cell index) automatically recorded by the RTCA system over the course of the entire experiment was plotted against time and presented.

### siRNA-Induced Knockdown of GSK3β and CRMP2

SMARTpool^®^ siRNA targeted against GSK3β, CRMP2 and non-specific control siRNA (Dharmacon ON-TARGETplus siRNA Reagents, Thermo Fisher Scientific) were used. Actively growing HuT78 and PBL T-cells (1.2×10^6^ cells) were mixed with siRNA molecules (100 nM) in the SF Cell Line and P3 Primary Cell 4D-Nucleofector™ X Kit, respectively. Cells were the nucleofected using the 4D-Nucleofector™ system (Lonza) according to the manufacturer’s instructions and used for experiments after 72 h.

### Co-Immunoprecipitation and Western Immunoblotting

T-cells treated under various experimental conditions were washed with PBS (4°C) and lysed in the cell lysis buffer as described earlier (15). The protein content of the cell lysates was determined by the Bradford protein assay (Bio-Rad). For co-immunoprecipitation assays, whole cell lysates (WCL, 500 µg each) were gently mixed with 3 µg of the target antibody or an isotype control IgG. Protein A/G plus agarose beads (25 µl/sample) were added to the antibody/cell lysate mix and incubated for 4 h at 4°C on a benchtop rotating/rocking shaker. The immune complexes were gently washed with the buffer containing 0.1% Triton X-100, 20 mM HEPES (pH 7.4), 130 mM NaCl, 10% glycerol, 1 mM phenylmethylsulfonyl fluoride, 10 mM sodium fluoride, 2 mM sodium vanadate and a cocktail of protease inhibitors. WCL or immunoprecipitated protein samples were heated in Laemmli sample buffer (95°C for 5 min), separated by gel electrophoresis, and then transferred to a nitrocellulose or PVDF membrane. Membranes were blocked using 5% Blotto or 2.5% bovine serum albumin (BSA) (Thermo Fisher Scientific) in PBS-0.05% Tween 20 for about 1 h at room temperature. Membranes were then incubated with primary antibody overnight at 4°C on a rotating shaker. After three washes, membranes were probed with corresponding HRP-conjugated secondary antibody for 1-2 h at room temperature. After washing, membranes were developed using an enhanced chemiluminescence reagent (Thermo Fisher Scientific) and imaged using ChemiDoc™ Gel Imaging System (Bio-Rad) or light sensitive films.

### GSK3β Interactome Analysis by LC-ESI-MS/MS

GSK3β-interacting proteins were co-immunoprecipitated from cellular lysates of resting (unstimulated) or LFA-1/ICAM-1-stumulated migrating T-cells using anti-GSK3β antibody and peptide identification was carried out by LC-MS/MS analysis. Briefly, GSK3β co-immunoprecipitated samples (from 2 mg protein each) were resolved by native gel electrophoresis and the proteins were digested in-gel with trypsin (Promega) after reduction and alkylation. Tryptic peptides were desalted using a C18 SPE cartridge (Waters, Singapore). The peptides were dried, reconstituted with 3% acetonitrile and 0.1% formic acid, and then separated and analysed using a coupled to a Q-Exactive tandem mass spectrometry coupled with Dionex Ultimate™ 3000 RSLCnano system (Thermo Fisher Scientific). Separation was performed on an EASY-Spray™ column (75 µm × 10 cm) packed with PepMap C18 3 µm, 100 Å (Thermo Fisher Scientific) using solvent A (0.1% formic acid) and solvent B (0.1% formic acid in 100% acetonitrile) at flow rate of 300 nL/min with a 60 min gradient. Peptides were then analysed on the Q-Exactive apparatus with the EASY-Spray™ Source (Thermo Fisher Scientific) at an electrospray potential of 1.5 kV. A full MS scan (350–1,600 m/z range) was acquired at a resolution of 70,000 and a maximum ion accumulation time of 100 ms. Dynamic exclusion was set as 30 s. The resolution of the higher energy collisional dissociation spectra was set to 350,00. The automatic gain control settings of the full MS scan and the MS2 scan were 5E6 and 2E5, respectively. The 10 most intense ions above the 2,000-count threshold were selected for fragmentation in higher energy collisional dissociation, with a maximum ion accumulation time of 120 ms. An isolation width of 2 m/z was used for MS2. Single and unassigned charged ions were excluded from MS/MS analysis. For higher energy collisional dissociation, the normalized collision energy was set to 28. The underfill ratio was defined as 0.3%. Raw data files were processed and converted to Mascot generic files format and submitted for database searching against the UniProt Human database with Mascot (v2.4.1, Matrix Science). The criteria used to filter results included 1% false positive threshold, expect value < 0.05 for significant peptide matches and the emPAI score was calculated as per a standard Mascot protein family report. Moreover, identification of peptides required at least two unique peptides under the standard search parameters. The mascot results were exported as.csv files for further analysis in Excel program (Microsoft Singapore Pte Ltd.).

### Ingenuity Pathway Analysis (IPA^®^)

The IPA^®^ software program (Qiagen) is a well-established bioinformatics tool facilitating identification of molecular relationships, mechanisms, and functions through dynamic pathway modelling. An updated repository of biological interactions (Ingenuity^®^ Knowledge Base) is utilized to create functional annotations from individually modelled relationships among proteins, genes, cells etc. We employed IPA^®^ to decipher dynamic molecular changes in GSK3β protein-protein interactions between resting T-cells and LFA-1/ICAM-1-stimulated migrating T-cells. To generate biological networks, protein dataset obtained from Mascot analysis was uploaded onto the IPA^®^ software and IPA^®^ protein networks were created and scored based on a Fisher’s exact test, indicating the likelihood of proteins associating into the GSK3β network by random chance. The core analysis was restricted to the immune cells to extract the relationships.

### Confocal Microscopy, High Content Imaging and Analysis

T-cells were allowed to migrate on rICAM-1-coated (migrating) or PLL-coated (resting control) coverslips for 2 h and then cells were fixed with 4% (v/v) formaldehyde for 10 min as described (11). After permeabilization using 0.3% Triton X-100 (prepared in PBS) and blocking in 5% BSA, cells were immunostained for selected proteins. Hoechst-33342 was used to stain the nuclei. Fluorescently stained cells on coverslips were then mounted onto clear glass slides with the help of the Fluoromount™ Aqueous Mounting Medium (Sigma-Aldrich). A Zeiss LSM800 Airyscan microscope attached with 405, 488, 561, and 647 nm lasers and a 63X/1.4 numerical aperture (NA) oil immersion objective lens (Carl Zeiss, Inc.) was used for confocal imaging. At least 3 images were acquired under each treatment condition and ZEN lite 2.1 (Carl Zeiss) software was used for image processing, analysis, and presentation. Intensity profiles of selected molecular signals in the confocal images were generated using the ZEN lite 2.1 and were replotted using the GraphPad Prism software. To quantify the colocalization of the CRMP2 and pericentrin proteins, Pearson Correlation Coefficient (PCC) was calculated using the ZEN Black software (Carl Zeiss). Cellular/nuclear location of GSK3β, pGSK3β-S9, and CRMP2 in motile T-cells was quantified by high content imaging and automated analysis. Briefly, T-cells were allowed to migrate on the wells of the rICAM-1-coated 96-well tissue culture plate (2×10^4^ cells/per well) for multiple time-points up to 2 h and fixed. Cells were then fluorescently labelled for GSK3β, pGSK3β-S9 or CRMP2 and co-stained with Rhodamine-Phalloidin and Hoechst to demarcate cytoplasmic and nuclear regions. Fluorescently labelled cells were then imaged by an automated microscope IN Cell Analyzer 2200 (GE Healthcare) using 20X objective (6 fields/well). Acquired image-sets containing >500 cells/well were subsequently analyzed cell-by-cell using the IN Cell Investigator software.

### Statistical Analysis

The level of statistical significance was computed using one-way analysis of variance (ANOVA) with Dunnett’s correction among experimental groups and the t-test using GraphPad Prism (v8.4.3, GraphPad). Difference with p < 0.05 was considered as significant.

## Results

### GSK3β Inhibition Promotes T-Cell Motility

**Figure 1:**
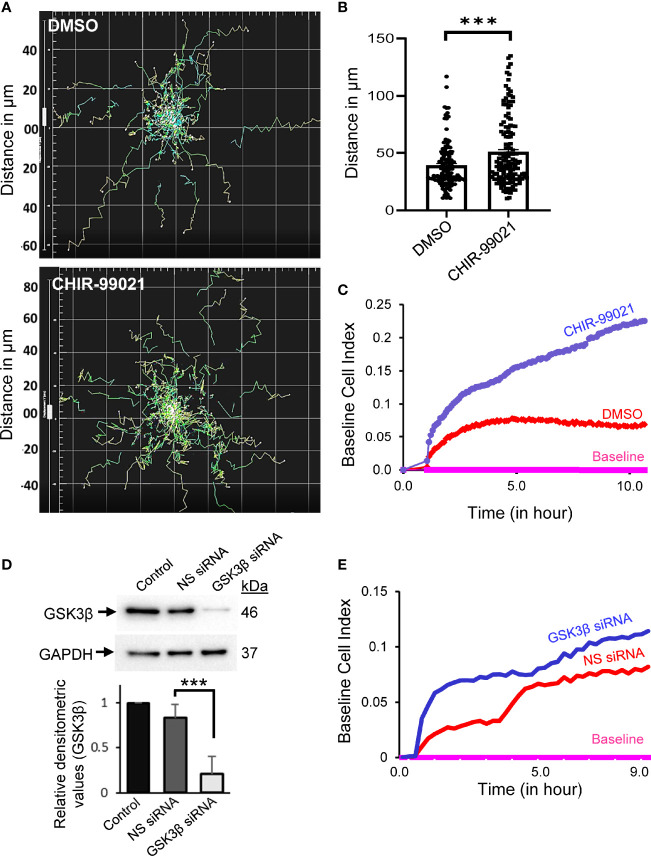
Inhibition or depletion of GSK3β enhances T-cell motility. **(A)** Human primary T-cells were pre-treated with 5 µM CHIR-99021 for 2 h to inhibit GSK3β or DMSO (solvent control). Cells were then allowed to migrate on rICAM-1-coated plate and tracked in live cell microscopy. Spider plots showing the traced tracks of cells are presented. **(B)** Distance travelled by migrating T-cells over a 2-h period in μm. **(C)** Transwell chemotaxis of primary T-cells towards the chemokine SDF-1α, as determined using CIM-Plate 16 and real-time impedance-based measurements by the RTCA instrument. **(D)** HuT78 T-cells were nucleofected with 100 nM siRNA targeting GSK3β or non-specific (*NS*) siRNA. After 72 h, cells were lysed and the expression levels of GSK3β was determined by Western immunoblotting. Blots were re-probed for GAPDH as a loading control. The relative densitometry values for GSK3β were determined and plotted (mean ± SEM). **(E)** Transwell chemotaxis of control (*NS siRNA*) and GSK3β-depleted (*GSK3β siRNA*) HuT78 T-cells towards SDF-1α was determined using CIM-Plate 16 and real-time impedance-based measurements. Baseline was drawn automatically for wells without SDF-1α. Data represent at least three independent experiments. ***p < 0.001.

We first investigated the involvement of GSK3β in T-cell migration by real-time monitoring of motile T-cells in the presence of an established GSK3β inhibitor, CHIR-99021, using an automated live cell imaging. CHIR-99021 specifically inhibits GSK3α/β and its IC_50_ concentration is 7-10 nM in cell-free *in vitro* assays (16, 17). The effective inhibitory concentrations of CHIR-99021 in cultured mammalian cells have been reported to be in the range of 3 to 10 µM (18–31). Here we choose to pre-treat T-cells with 5 µM CHIR-99021 for 2 h to inhibit GSK3β in our experiments. Cellular treatment with CHIR-99021 significantly enhanced the migratory behaviour of T-cells following stimulation by LFA-1/ICAM-1 engagement (**Figure 1A** and **Videos 1**, **2** in **Supplementary Material**) without impacting T-cell viability (**Supplementary Figure S1A** in **Supplementary Material**). Quantification of the trajectories taken by motile T-cells over the course of 2 h showed that CHIR-99021-treated T-cells travelled significantly (>20%) longer distance compared to control (**Figure 1B**). GSK3β inhibition significantly increased the chemotactic potential of motile PBL T-cells as analysed by transwell assay using real-time impedance-based measurements (**Figure 1C**). Similarly, siRNA-induced knockdown of GSK3β in HuT78 T-cells (**Figure 1D**) enhanced their migratory action (**Figure 1E**) without impacting cell viability (**Supplementary Figure S1B** in **Supplementary Material**). Notably, CHIR-99021 treatment did not impact the ability of T-cells to proliferate or produce cytokines (IL-2 and IFN-γ) in response to activation *via* the T-cell receptor (**Supplementary Figure S2** in **Supplementary Material**).

### GSK3β Interactome in LFA-1-Stimulated Migrating T-Cells Identifies Notch1 and CRMP2 Interactions

**Figure 2:**
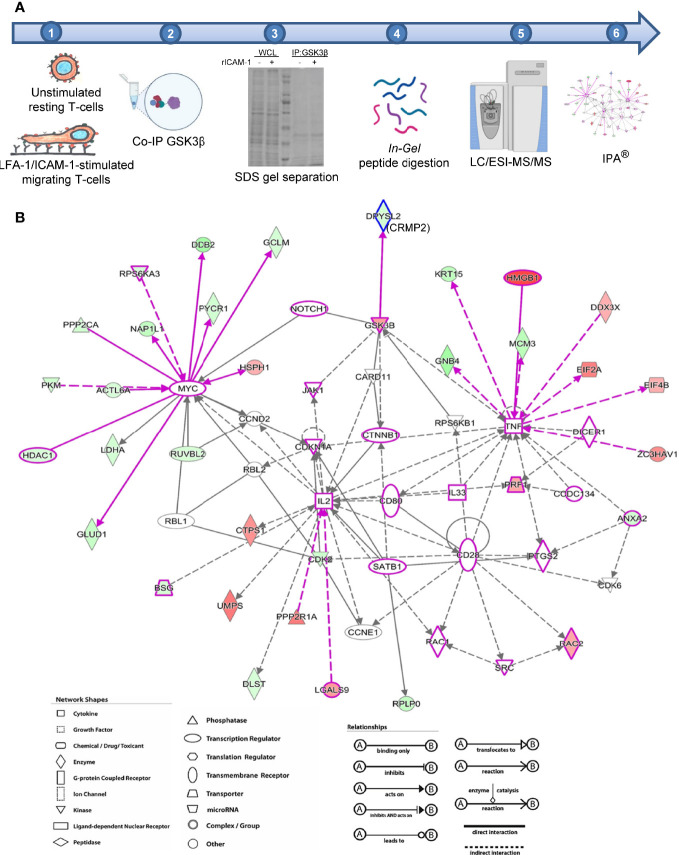
Identification of GSK3β interactome in migrating T-cells. **(A)** Schematic representation of workflow employed in mass spectrometry-based proteomics analysis of GSK3β interacting proteins in human HuT78 T-cells. **(B)** Image output from IPA Ingenuity Knowledge Base indicating statistically probable interactions of GSK3β in LFA-1-stimulated T-cells. Proteins depicted in red are high abundance in migrating T-cells and proteins shown in green are low in abundance, compared to resting T-cells. The protein symbols in purple highlight proteins curated from Ingenuity Knowledge Base with potential roles in cellular movement and modelled relationships among known functional networks in immune cells. The type of proteins, network, shapes, and relationships in IPA are provided in the legends.

We next determined intracellular proteins that interact with endogenous GSK3β in LFA-1-stimulated motile T-cells by co-immunoprecipitation with anti-GSK3β antibody and subsequent mass spectrometry analysis as illustrated in **Figure 2A**. We detected 1,168 unique proteins directly or indirectly pulled down by anti-GSK3β [Mascot protein database search against Human Uniprot protein database, false discovery rate (FDR) ≤1%]. Based on protein abundance exponentially modified Protein Abundance Index (emPAI) scores (32) of GSK3β interactome, 256 candidate proteins were identified to be differentially associated with GSK3β (82 protein IDs with ≥2-fold higher emPAI score and 174 protein IDs with ≥2-fold lower emPAI score in LFA-1/ICAM-1-stimulated migrating T-cells compared to unstimulated resting cells, **Supplementary Table 1**). Of the 256 protein ID’s, 243 were “analysis ready” consistent with Ingenuity^®^ Knowledge Base and generated multiple canonical pathways, upstream regulators, associated diseases, and cellular functions. The top diseases and functions associated with the GSK3β interactome included cellular compromise, cellular movement, inflammatory response, and immune cell trafficking. Of the major canonical pathways with a positive Z-score among the protein networks were RhoGDI signaling, sirtuin signaling pathway, hippo signaling and Wnt/β-catenin signaling pathways (**Supplementary Figure S3** in **Supplementary Material**). Four direct interactions of GSK3β identified in the enriched network were *i*) Notch1, *ii*) dihydropyrimidinase-related protein 2 (DPYSL2, also called CRMP2), *iii*) ribosomal protein S6 kinase beta-1 (RPS6KB1), and *iv*) caspase recruitment domain-containing protein 11 (CARD11) (**Figure 2B**). Of note, based on empirical abundance scores, the CRMP2-GSK3β association was more pronounced in resting T-cells in comparison to migrating T-cells.

### GSK3β Interaction With Notch1 and CRMP2 in LFA-1/ICAM-1-Stimulated Motile T-Cells

**Figure 3:**
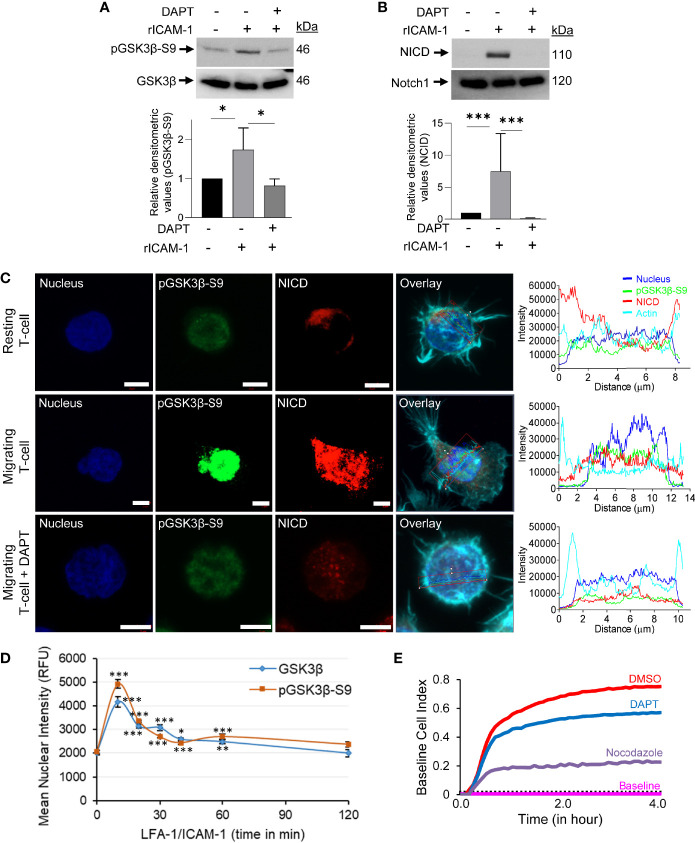
GSK3β-Notch1 interactions in motile T-cells. **(A, B)** Human primary T-cells, untreated or pre-treated with 10 μM DAPT, were stimulated to migrate on rICAM-1-coated plates for 30 min and lysed. Cellular lysates were Western immunoblotted for pGSK3β-S9, GSK3β, NICD and total Notch1. The expression levels of proteins were quantified by determining relative densitometry (mean ± SEM) of pGSK3β-S9/GSK3β and NICD/Notch1. **(C)** Resting and LFA-1-stimulated HuT78 T-cells were immunostained with anti-pGSK3β-S9/Alexa Fluor^®^ 488 (*green*), anti-NICD/Alexa Fluor^®^ 568 (*red*), Phalloidin-Alexa Fluor^®^ 647 (actin, *cyan*) and Hoechst (nucleus, *blue*) and then imaged by confocal laser scanning microscopy, 63X oil objective. Overlay images with intensity profiles (replotted using the GraphPad Prism software) of nucleus, pGSK3β-S9, NICD and actin are shown. Scale bar = 5 μm. **(D)** T-cells (2×10^4^ cells/per well) were allowed to migrate on rICAM-1-coated 96-well plate for multiple time-points up to 2 h and fixed. Cells were stained for pGSK3β-S9 or GSK3β and imaged by an automated IN Cell Analyzer 2200 microscope using 20X objective (6 fields/well). Cellular images containing >500 cells/well were subsequently analysed cell-by-cell by the IN Cell Investigator software. **(E)** HuT78 T-cells were pre-treated with 10 µM DAPT, nocodazole (positive control), or DMSO (solvent control) and then allowed to migrate on rICAM-1-coated E-Plate 16. Cell migration was recorded in real-time using impedance-based measurements by the RTCA instrument. Wells without cells were used to automatically draw the baseline. Data represent at least three independent experiments. *p < 0.05; **p < 0.01; ***p < 0.001 compared to corresponding resting T-cells (0 min) control; RFU, relative fluorescent intensity.

Concurrent with an increased phosphorylation of GSK3β (pGSK3β-S9) and cleavage of Notch1 intracellular domain (NICD) following LFA-1 stimulation (**Figures 3A, B**), pGSK3β-S9 translocated to the nucleus in LFA-1/ICAM-1-stimulated migrating T-cells (**Figure 3C** and **Supplementary Figure S4** in **Supplementary Material**). The LFA-1-induced translocation of pGSK3β-S9 to the nucleus occurred in a time-dependent manner reaching to maximum in a time-window of 10 to 30 min of stimulation (**Figure 3D**). This pGSK3β-S9 translocation was accompanied with cleaved NICD in motile T-cells (**Figure 3C**). Blocking Notch1 cleavage by the γ-secretase inhibitor DAPT (**Figure 3B**) inhibited LFA-1-induced pGSK3β-S9 phosphorylation (**Figure 3A**) and its nuclear translocation (**Figure 3C**, middle vs lower panels), and reduced T-cell migration (**Figure 3E**).

**Figure 4:**
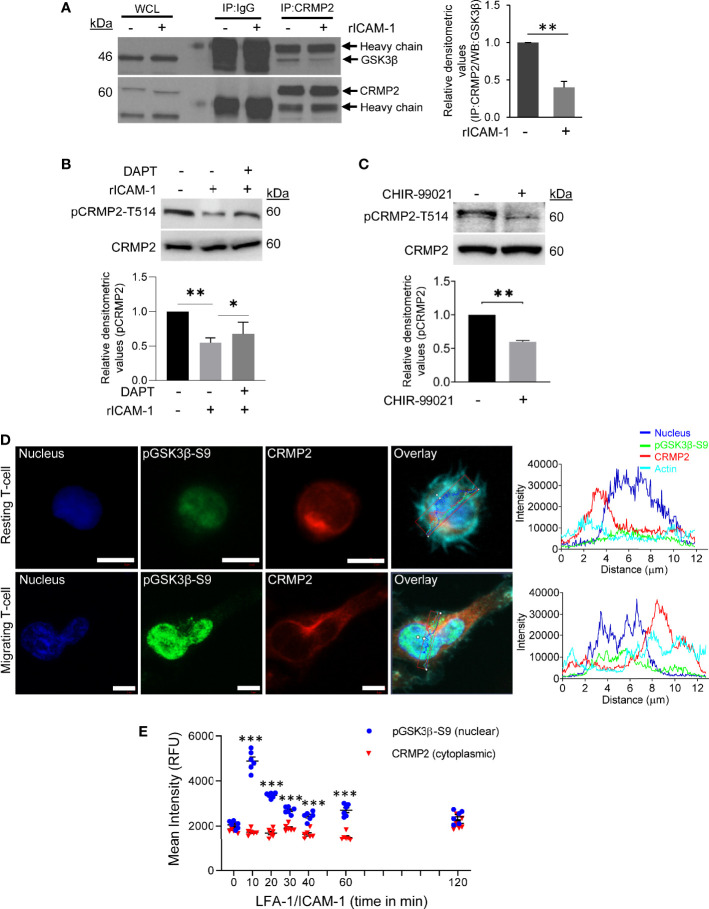
GSK3β-CRMP2 interactions in motile T-cells. **(A)** Human primary T-cells were stimulated to migrate on rICAM-1-coated plates for 30 min and lysed. Cellular lysates from unstimulated or LFA-1/ICAM-1-stimulated T-cells were immunoprecipitated (*IP*) with anti-CRMP2 and Western immunoblotted for GSK3β and CRMP2. The amounts of GSK3β co-precipitating with CRMP2 were quantified by densitometry analysis (mean ± SEM). **(B)** Human primary T-cells, untreated or pre-treated with 10 μM DAPT, were stimulated to migrate on rICAM-1-coated plates for 30 min and lysed. **(C)** Human primary T-cells were treated with 5 μM CHIR-99021 or an equivalent amount of DMSO (solvent control) for 2 h and lysed. Cellular lysates in “B” and “C” were Western immunoblotted for pCRMP2-T514 and total CRMP2. The relative expression levels of pCRMP2-T514 were quantified by densitometry analysis (mean ± SEM). **(D)** Resting and LFA-1-stimulated migrating HuT78 T-cells were immunostained with anti-pGSK3β-S9/Alexa Fluor^®^ 488 (*green*), anti-CRMP2/Alexa Fluor^®^ 568 (*red*), Phalloidin-Alexa Fluor^®^ 647 (actin, *cyan*) and Hoechst (nucleus, *blue*), and then imaged by confocal laser scanning microscopy, 63X oil objective. Overlay images with intensity profiles (replotted using the GraphPad Prism software) of nucleus, pGSK3β-S9, CRMP2 and actin are shown. Scale bar = 5 μm. **(E)** T-cells (2×10^4^ cells/per well) were allowed to migrate on rICAM-1-coated 96-well plate for multiple time-points up to 2 h and fixed. Cells were stained for CRMP2 or pGSK3β-S9 and imaged by an automated IN Cell Analyzer 2200 microscope using 20X objective (6 fields/well). Cytoplasmic and nuclear intensities of CRMP2 and pGSK3β-S9, respectively, from >500 cells/well were subsequently analysed cell-by-cell by the IN Cell Investigator software. Each dot represents mean intensity values from >500 cells. Data represent at least three independent experiments. *p < 0.05; **p < 0.01; ***p < 0.001 compared to resting T-cells (0 min) control; RFU, relative fluorescent intensity.

To comprehend an interaction between GSK3β and CRMP2, T-cells were stimulated to migrate *via* LFA-1/ICAM-1 and lysed. Cellular lysates were then immunoprecipitated using an anti-CRMP2 antibody or IgG (control) and probed for GSK3β and CRMP2. Consistent with protein abundance scores from mass spectrometry, immunoblotting revealed a decreased interaction between GSK3β and CRMP2 in LFA-1-stimulated T-cells in comparison to unstimulated resting cells (**Figure 4A**). LFA-1 stimulation resulted in decreased phosphorylation of CRMP2 at the Thr514 residue, which could be partially rescued by DAPT (**Figure 4B**). The decreased phosphorylation of CRMP2 was also evident in T-cells treated with the GSK3β inhibitor CHIR-99021 (**Figure 4C**). While pGSK3β-S9 translocated to the nucleus following LFA-1 stimulation, CRMP2 remained in the cytoplasm (**Figures 4D, E** and **Supplementary Figure S5** in **Supplementary Material**).

**Figure 5:**
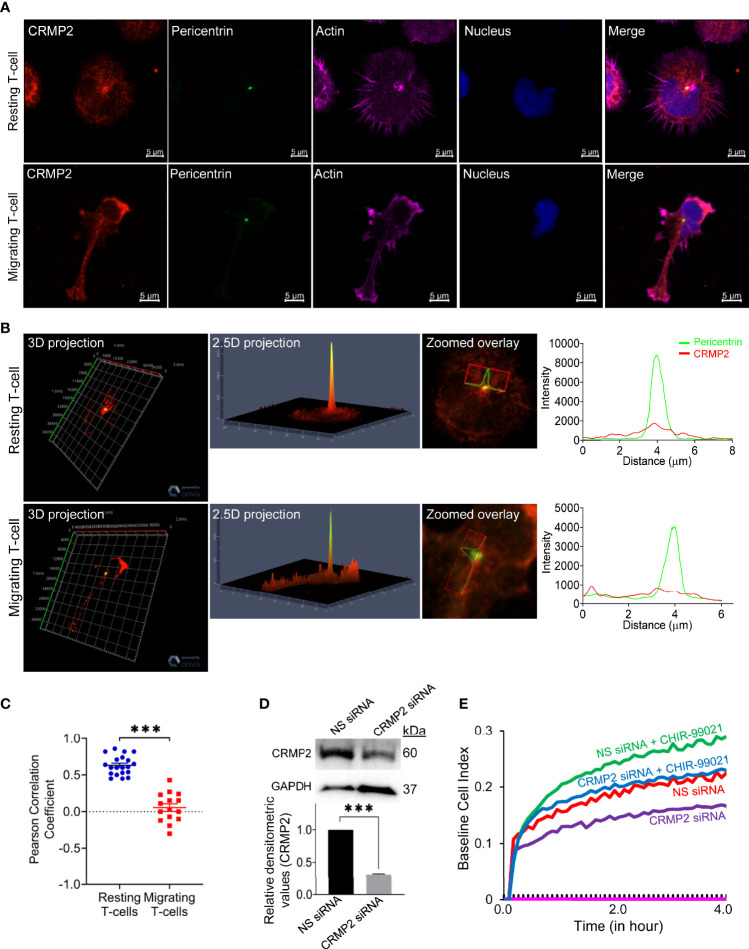
CRMP2 colocalization to the MTOC and the effect of CRMP2 depletion on T-cell motility. **(A)** Resting and LFA-1-stimulated HuT78 T-cells were immunostained with anti-CRMP2/Alexa Fluor^®^ 568 (*red*), anti-pericentrin/Alexa Fluor^®^ 488 (*green*), phalloidin-Alexa Fluor^®^ 647 (actin, *pink*) and Hoechst (nucleus, *blue*). Cells were then imaged by confocal laser scanning microscopy, array scan 63X objective. **(B)** 3D and 2.5D projections, zoomed overlay and intensity profiles (replotted using the GraphPad Prism software) of CRMP2 and pericentrin are shown. **(C)** Pearson Correlation Coefficient between CRMP2 and pericentrin was assessed using Carl Zeiss ZEN Black software. Each dot represents a single T-cell, and the images were taken from at least three independent experiments; n=20 for resting T-cells and n=15 for migrating T-cells; error bar, mean ± SEM. **(D)** PBL T-cells were nucleofected with 100 nM siRNA targeting CRMP2 or non-specific (*NS*) siRNA. After 72 h, cells were lysed, Western immunoblotted and probed for CRMP2. Blots were re-probed for GAPDH as a loading control. Relative densitometry values for CRMP2/GAPDH were determined and plotted (mean ± SEM). **(E)** The control (*NS siRNA*) and CRMP2-depleted (*CRMP2 siRNA*) PBL T-cells were treated with 5 µM CHIR-99021 for 2 h and then allowed to migrate on rICAM-1-coated E-Plate 16 for 4 h. Cell migration was recorded in real-time using impedance-based measurements by the RTCA instrument. Wells without cells were used to automatically draw the baseline. Data represent at least three independent experiments. ***p < 0.001.

To examine cellular localization of CRMP2 in motile T-cells, we performed confocal microscopy and subsequent image analysis. In resting T-cells, substantial amounts of CRMP2 were localized in close proximity to the microtubule-organizing center (MTOC), as determined by co-staining of cells with pericentrin (an MTOC marker) (**Figures 5A, B**). We used PCC to assess the overall proximity of CRMP2 and MTOC, additional confirmation of colocalization that provides quantitative values ranging from +1.0 (total positive correlation), 0 (no correlation) to -1.0 (total negative correlation). The mean PCC value for CRMP2 and pericentrin in unstimulated resting T-cells was above 0.5 (**Figure 5C**), indicating a high instance of colocalization. This MTOC colocalization of CRMP2 was lost following LFA-1 stimulation in motile T-cells with the mean PCC value significantly reduced to less than 0.1 (**Figure 5C**), indicating no-to-low colocalization. No colocalization or containment of CRMP2 with the Golgi was detected in either resting or LFA-1-stimulated T-cells (**Supplementary Figure S6** in **Supplementary Material**). No further change in LFA-1-induced Notch1 cleavage or CRMP2 Thr514 phosphorylation was observed in cells that were pre-incubated with CHIR-99021 (**Supplementary Figure S7** in **Supplementary Material**). Most importantly, the migratory advantage conferred by CHIR-99021 treatment was lost when CRMP2 expression was knocked-down in T-cells (**Figures 5D, E**), indicating that GSK3β inhibition favours CRMP2-dependent T-cell migration. CRMP2-depleted cells exhibited an inhibition of migration compared to control T-cells (**Figure 5E**), implying a crucial involvement of CRMP2 in T-cell motility.

## Discussion

The current study demonstrates a crucial involvement of GSK3β in T-cell migration, which is important for T-cells to respond to environmental cues, such as chemokines, in order to mount an effective immune response. We show that selective inhibition or depletion of GSK3β enhances T-cell motility. GSK3β putatively interacts with multiple proteins in the signaling pathways triggered *via* LFA-1/ICAM-1 engagement in motile T-cells. In particular, GSK3β interacts with Notch1 and CRMP2 during the process of T-cell locomotion.

CHIR-99021 is the most selective commercially available ATP-competitive inhibitor of GSK3α/β (16, 17). Cellular treatment with CHIR-99021 significantly increased the rate of migration and chemotaxis in human primary T-lymphocytes. In contrast, a recent study using a different inhibitor of GSK3α/β, SB415286, showed that prolonged (7 days) inhibition of GSK3 in mouse T-cells reduced cell motility in the presence of target cells (EL4-OVA) and decreased the number of cell-to-cell contacts required to induce target killing (33). At the same time, long-term inactivation of GSK-3α/β by SB415286 enhanced the tumor killing potency of the resultant cytolytic mouse T-cells (33, 34). Variable degree of inhibition of GSK3α/β due to inhibitor dose or discrepancies in period of culture could be assumed as the possible explanations for such divergent phenotypes. These variations also suggest a species-specific role of GSK3β in T-cell signaling and functioning. It has also been reported that the biological effects of CHIR-99021 are counteracted by the inhibition of Notch signaling (30).

GSK3β is one of the downstream substrates of the nutrient-sensing kinase, AMP-activated protein kinase (AMPK). This kinase is important in promoting glucose uptake and ATP production to match energy demands in motile T-cells in response to chemokines, such as CCL5 (35). Dynamic interactions among GSK3β, AMPK and β-catenin have been found to be important in controlling metabolic reprogramming, migration, and invasion in anoikis-resistant prostate cancer cells (36). Moreover, in a cultured glioma cell line U-251, GSK3β was shown to interact with Akt and snail and CUX1 pathway and regulate ionizing radiation-induced epithelial-mesenchymal transition as well as migration and invasion (37). In mice, an impaired inducible inactivation of the GSK3β, due to the loss of mDia1 and diminished microtubule dynamics, has been associated with compromised T-cell adhesion, migration, and *in vivo* trafficking (38). A regulatory role of GSK3β in the migration of high-glucose-induced human skin fibroblasts and neural crest lineage in mouse and Xenopus has also been reported (39, 40).

Notch1 is an important substrate for GSK3β (41) and plays a role in T-cell homeostasis and differentiation (42, 43). At the same time, GSK3β functions positively within the Notch pathway and protects the NICD from proteasome-mediated degradation (44). Notch signaling was found to be impaired in GSK3β-null embryonic fibroblasts (44). We have earlier reported GSK3β as an upstream regulator of Notch1 in the LFA-1 signaling pathway leading to T-cell Th1 polarization (9). In the present study, we showed that DAPT, in addition to inhibiting Notch1 cleavage, inhibited LFA-1-induced GSK3β Ser9 phosphorylation and its nuclear translocation. These suggest that GSK3β is also positioned downstream of Notch1 in the LFA-1-induced signaling cascade during the process of T-cell locomotion. We argue that the dynamic and interactive nature of LFA-1-stimulated signaling cascades dictate that downstream signals are in receipt of multiple communications at any given time. T-cell motility represents a net response to these intracellular signals and therefore the pathways mediating LFA-1 responses are frequently integrated. Simultaneous triggering of multiple pathways by LFA-1 stimulation could coincide with GSK3β Ser9 phosphorylation in potentiating Notch-dependent responses. We could also argue that bidirectional interactions exist between GSK3β and Notch pathways. Since GSK3β prefers prior phosphorylation of its substrates (45), NICD is likely to be primed by other kinases that are concurrently activated following LFA-1 stimulation. For example, the cyclin-dependent kinase 8 (Cdk8), Cdk5, and the dual-specificity tyrosine-regulated kinase 2 are known to phosphorylate NICD in various cell types (46–48). Earlier genetic studies using the Drosophila GSK3 ortholog, shaggy, and the rat GSK3 isoforms placed GSK3β downstream of the Notch in the transmission of intracellular signals and upstream of the Notch in the regulation of a cell’s ability to communicate (49). These suggest that GSK3β integrates cell’s signal transmitting and receiving abilities and that Notch1 exerts its influence on GSK3β, a kinase known to phosphorylate and regulate Notch signals. It would therefore be interesting to explore whether LFA-1 signaling-induced Notch1 cleavage primes subsequent interactions between NICD and pGSK3β-Ser9 or GSK3β Ser9 phosphorylation occurs during interaction with NICD with potential feedback loops that stimulate Notch-1 activity in motile T-cells.

Of the four direct relationships observed in the GSK3β interactome, CARD11 and RPSK6B1 regulate antigen-induced lymphocyte activation and signaling relays involving the mTOR pathway (50, 51). Studies suggest a correlation between GSK3β and mTORC1 in the regulation of energy-reliant transcriptional networks by mitogenic or metabolic signals like PI3K-Akt or ATP (52). In response to chemotactic stimulation, GSK3 directly phosphorylates RacE-GDP at the Ser192 residue, which controls mTORC2-mediated phosphorylation of Akt and directed cell migration (53). In this context, further exploration of GSK3β interaction with CARD11, RPSK and mTOR pathways would provide vital inputs on energy-dependent mechanisms in T-cell motility. The proteomics database presented in this study thus provides a foundation for more detailed studies to uncover GSK3β involvement in T-cell migration.

CRMP2 (also known as CRMP-62, Ulip2, TOAD-64 and DRP-2), initially reported exclusively in the developing nervous system, plays an important role in specifying axon/dendrite fate, possibly by promoting neurite elongation *via* microtubule assembly. This protein was later found to be expressed in peripheral T-cells and involved in T-cell polarization, recruitment and neuroinflammation (54–57). In particular, the upregulation of CRMP2 expression was recorded in subsets of T-cells bearing early and late activation markers, CD69^+^ and HLA-DR^+^, respectively (55). An involvement of CRMP2 in T-cell migration mediated *via* the chemokine CXCL12 (SDF-1α) and the extracellular signaling protein semaphorin has also been reported earlier (55, 56). In addition, previous studies noted a polarized distribution of CRMP2 at the uropod and its binding to the cytoskeletal protein, vimentin, following CXCL12-induced signaling (55, 56). In the current study, we observed substantial amounts of CRMP2 localized to the MTOC in resting T-cells, which was lost following LFA-1 stimulation in motile T-cells. These findings further confirm a role of CRMP2 in dynamic remodeling of the cytoskeletal systems during T-cell motility.

CRMP2 has been described as a microtubule-associated protein (58) that regulates microtubule dynamics in multiple ways. It associates with α/β-tubulin heterodimers and promotes their transport to the plus end of the growing microtubule (59). It serves as an adaptor to bring together motor proteins (e.g., kinesin-1) and tubulins to promote microtubule elongation (60). It enhances the GTPase activity of the β-tubulin and promotes the polymerization of α/β-tubulin heterodimers on the curved sheets of the microtubule ends (61). As microtubules elongate, CRMP2 moves along the growing plus end to stabilize newly polymerized microtubules (61). The phosphorylation of CRMP2 impedes the binding between CRMP2 and the microtubule (58, 62, 63). In neural cells, sequential phosphorylation of CRMP2 at the C-terminus by several serine/threonine kinases has been shown to be crucial for CRMP2 function (62). For example, Rho-kinase phosphorylates CRMP2 at Thr555 (64, 65) and the Cdk5 kinase phosphorylates CRMP2 at Ser522 (57, 66). Differential phosphorylation of CRMP2 at multiple sites by multiple kinases is thus a crucial regulatory mechanism for the dynamic reorganization of cytoskeleton required for the movement of different cell types. Structural studies have shown that the C-terminus phosphorylation of CRMP2 (*e.g.*, Thr514) confers negative charges adding repulsive forces between the CRMP2 and the E-hook of tubulin, that reduces its tubulin binding affinity and negatively regulates microtubule growth and stability, thus having the opposite effect of unphosphorylated CRMP2 (61, 67). CRMP2 dephosphorylation at Thr514 improves CRMP2 binding and stabilization of microtubules (63). In this regard, it can be inferred that observed decrease in CRMP2 Thr514 phosphorylation following LFA-1 stimulation or GSK3β inhibition by CHIR-99021 treatment promotes microtubule polymerization and facilitates T-cell migration. It would be fascinating to investigate, in future, whether decreased motility of CRMP2-depleted T-cells is due to microtubules being more susceptible to catastrophes in the absence of CRMP2.

In previous studies, Giraudon and colleagues reported CXCL12-induced decrease in CRMP2 phosphorylation at the Thr509/514 residues in motile T-cells (56). They further showed that this decrease in CRMP2 Thr509/514 phosphorylation was mediated *via* the GSK3β kinase (57). In addition, CXCL12 signaling was also found to enhance CRMP2 Tyr479 phosphorylation, a potential target site for the Src-family kinase Yes (56). It has been suggested that initial phosphorylation events in CRMP2 prime this protein for subsequent Thr509/514 phosphorylation by the GSK3β (68). In hippocampal neurons, inactivation of GSK3β by neurotrophin-3 was found to cause CRMP2 dephosphorylation leading to axon elongation and branching (63). Moreover, promotion of axonal regeneration was observed following genetic inhibition of CRMP2 phosphorylation at the Ser522 residue in a mouse model of optic nerve injury (69). Decreased interaction between GSK3β and CRMP2, diminished colocalization of CRMP2 with MTOC, and reduced CRMP2 phosphorylation (pCRMP2-T514) following LFA-1 stimulation and GSK3β inhibition by CHIR-99021 demonstrated in the current study provide a novel regulatory mechanism in T-cell motility.

Heightened CRMP2 expression in T-cell clones derived from patients that were infected with the retrovirus HTLV-1 has been associated with pathological T-lymphocyte CNS infiltration, implicated in virus-induced neuroinflammation (54, 57). The decreased interaction between GSK3β and CRMP2 facilitated by GSK3β Ser9 phosphorylation and NICD-GSK3β nuclear translocation observed in the current study could explain enhanced T-cell infiltration in neuroinflammation due to high levels of active CRMP2. Since multiple priming kinases and phosphatases contribute to differential regulation of CRMP2 by GSK3β (68), it is possible that, in addition to GSK3β, other enzymes are also activated by LFA-1/ICAM-1 cross-linking which phosphorylate/dephosphorylate CRMP2 in motile T-cells. In this context, ongoing interactions among GSK3β, Notch1, and CRMP2 are crucial in the maintenance of polarity and motility in human T-lymphocytes.

**Figure 6:**
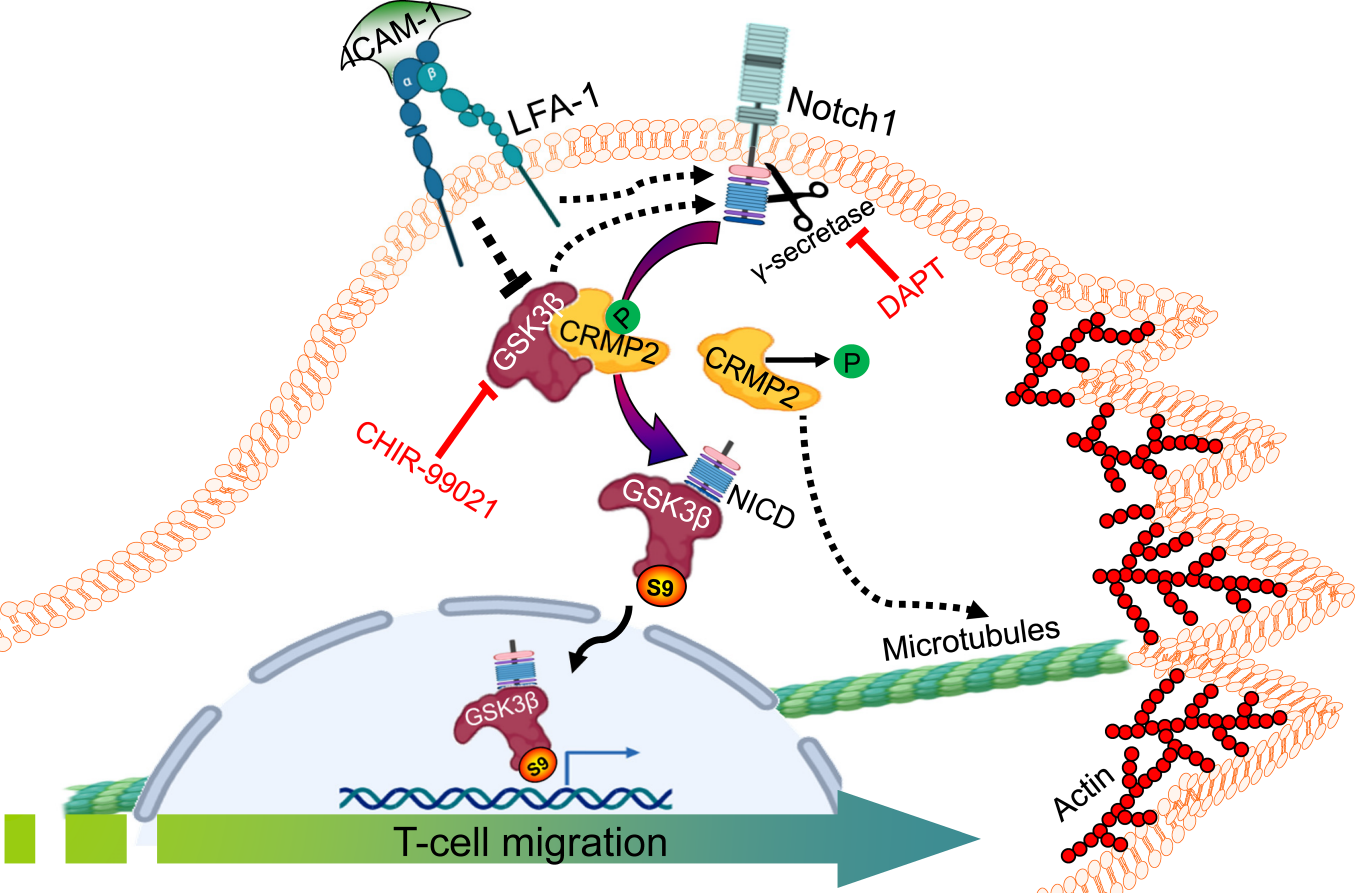
An illustration of GSK3β-Notch1 and GSK3β-CRMP2 interactions in T-cell motility. LFA-1 stimulation-mediated signals in motile T-cells inactivate GSK3β by inducing its Ser9 phosphorylation. pGSK3β-S9 interacts with cleaved NICD and translocates to the nucleus. CRMP2 released from bound GSK3β further coordinates T-cell motility. The image created with BioRender.com.

In conclusion, we demonstrate that LFA-1-induced Notch1 cleavage, GSK3β interaction with NICD and its inactivation by S9 phosphorylation (pGSK3β-S9), and consequent dephosphorylation of CRMP2 facilitate T-cell migration (**Figure 6**). Our work thus presents a novel mechanism involving GSK3β interaction with CRMP2 and Notch1 in the regulation of T-cell motility. These findings also imply that non-canonical GSK3β signaling plays a crucial role in the rapid response of T-cells to the extracellular signals. Targeting this multitier signaling interactions may therefore be considered to fine-tune T-cell motility, which has important implications in adaptive immune responses, chronic inflammation, and autoimmunity.

## Data Availability Statement

The original contributions presented in the study are included in the article/**Supplementary Material**. Further inquiries can be directed to the corresponding author.

## Author Contributions

NV conceptualized, designed, and supervised the project. MF, PP, BW, and AK performed experiments and contributed to the preparation of essential materials. NS performed GSK3β high content analysis experiments. MF, PP, and NV interpreted the results and drafted the manuscript. SS performed mass spectrometry and analysis, commented on the experiments, and edited the paper. All authors contributed to the article and approved the submitted version.

## Funding

This work was supported by the grants from the Singapore Ministry of Education (MOE) Academic Research Fund (AcRF) Tier 1 (2014-T1-001-141 and 2020-T1-001-062) and the National Research Foundation Singapore under its Open Fund Large Collaborative Grant (OFLCG18May-0028) and administered by the Singapore Ministry of Health’s National Medical Research Council (NMRC).

## Conflict of Interest

The authors declare that the research was conducted in the absence of any commercial or financial relationships that could be construed as a potential conflict of interest.

## Publisher’s Note

All claims expressed in this article are solely those of the authors and do not necessarily represent those of their affiliated organizations, or those of the publisher, the editors and the reviewers. Any product that may be evaluated in this article, or claim that may be made by its manufacturer, is not guaranteed or endorsed by the publisher.
