# Supplementary material for: GSK3β Interacts With CRMP2 and Notch1 and Controls T-Cell Motility
Source: Front Immunol. 2021 Dec 17;12:680071. doi: 10.3389/fimmu.2021.680071 (PMC8718691; doi:10.3389/fimmu.2021.680071)
Supplement: Supplementary file 1 [file DataSheet_1.pdf]

Supplementary Material

GSK3β Interacts with CRMP2 and Notch1 and Controls T-Cell Motility

Mobashar Hussain Urf Turabe Fazil, Praseetha Prasanan, Brandon Han Siang Wong, Amuthavalli Kottaiswamy, Nur Syazwani Binte Mohamed Salim, Siu Kwan Sze, Navin Kumar Verma

Supplementary Data

Movie 1. The trajectories of control T-cells migrating one rICAM-1-coated plate, shown in Figure 1A (DMSO).

Movie 2. The trajectories of CHIR-91002-treated T-cells migrating one rICAM-1-coated plate, shown in Figure 1A (CHIR-91002).

Supplementary Figure S1 – S7.

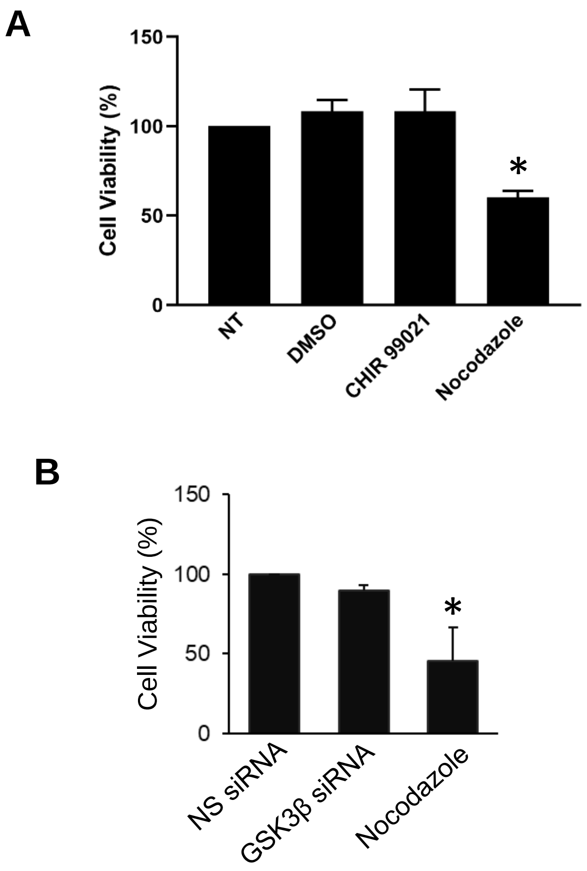

Supplementary Figure S1. Effect of CHIR-99021 or GSK3β siRNA on the viability of T-cells. **(A)** PBL T-cells (2×10^4 cells/per well) were plated in 96-well plates in triplicates and treated with 5 µM CHIR-99021 or equivalent amount of DMSO (solvent control) for 24 h. As negative or positive toxicity controls, cells were either left untreated (*NT*) or treated with 10 µM nocodazole. **(B)** HuT78 T-cells (1×10^5 cells) were nucleofected with 100 nM GSK3β siRNA or non-specific (*NS*) control siRNA and incubated for 48 h in triplicates. As a toxicity control, cells were treated with 10 µM nocodazole. Cell viability was determined using CellTiter 96® Aqueous One Solution. Absorbance (490 nm) readings were normalized and plotted as percentage (%) cell viability. Data is mean ± SEM of three independent experiments. *, $p<0.05$.

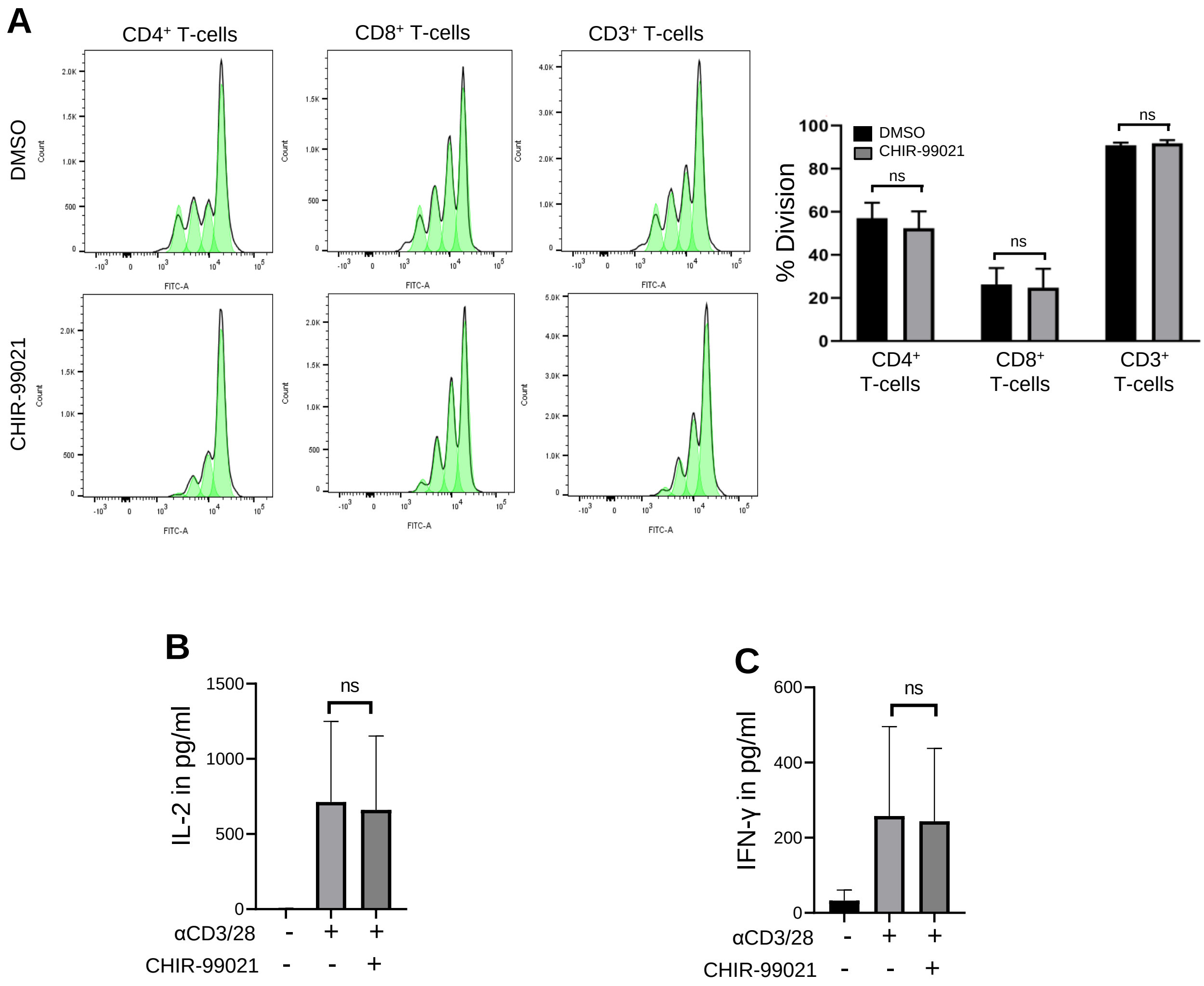

Supplementary Figure S2. Effect of GSK3β inhibition on T-cell proliferation and cytokine production. (**A**) PBL T-cells were labelled with the CFSE dye and activated *via* TCR by incubating on anti-CD3/CD28-coated plates for 72 h in the presence of 5 μM CHIR-99021 or DMSO. The proliferation of $CD3^+$, $CD4^+$ and $CD8^+$ T-cells was determined by flow-cytometry and plotted. (**B,C**) Secreted levels of IL-2 and IFN-γ were determined by ELISA. Data represent at least three independent experiments performed by using PBL T-cells derived from at least 3 different donors. Error bars represent mean ± SEM. *ns*, non-significant.

A. Canonical pathways

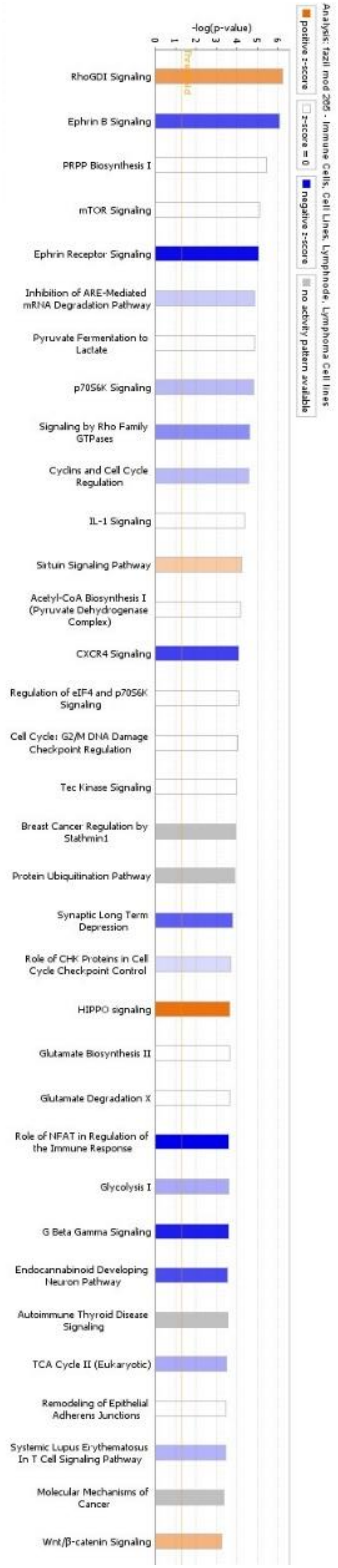

B. Diseases and functions of networks

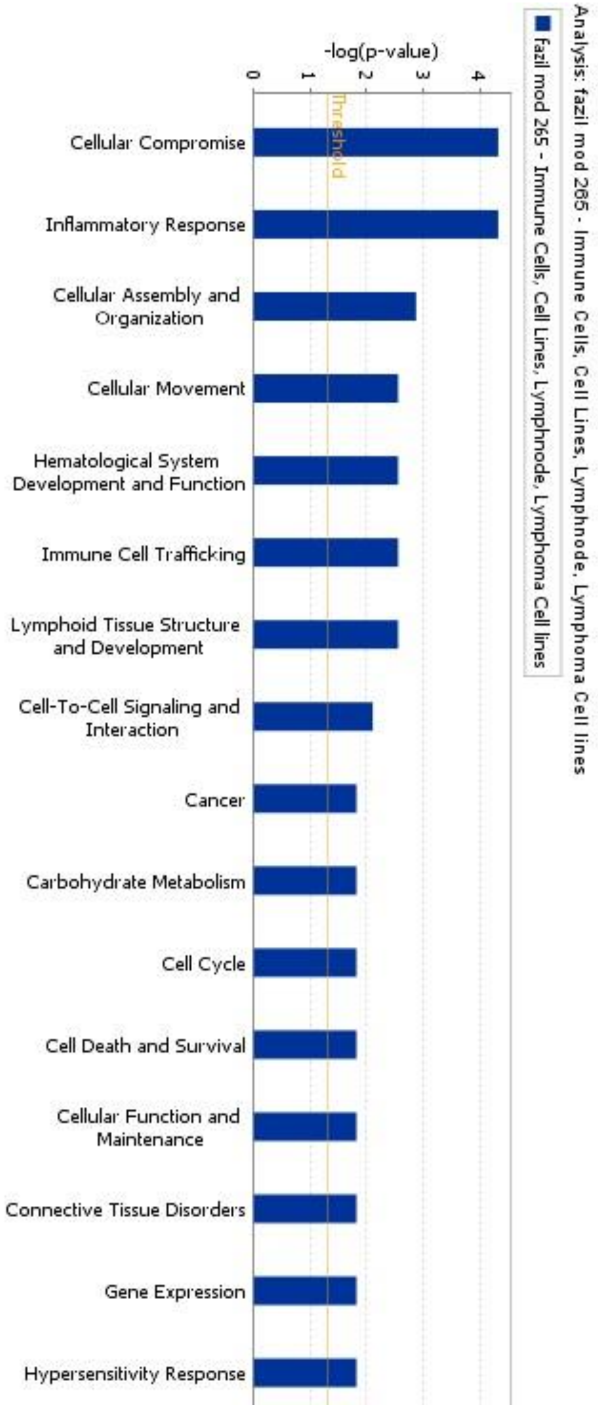

Supplementary Figure S3. IPA® analysis leveraged on Ingenuity® Knowledge Base reveals canonical pathways and disease functions of GSK3β interactome. (**A**) Canonical pathways and (**B**) diseases and functions of networks identified among the GSK3β interactome in motile T-cells by IPA®.

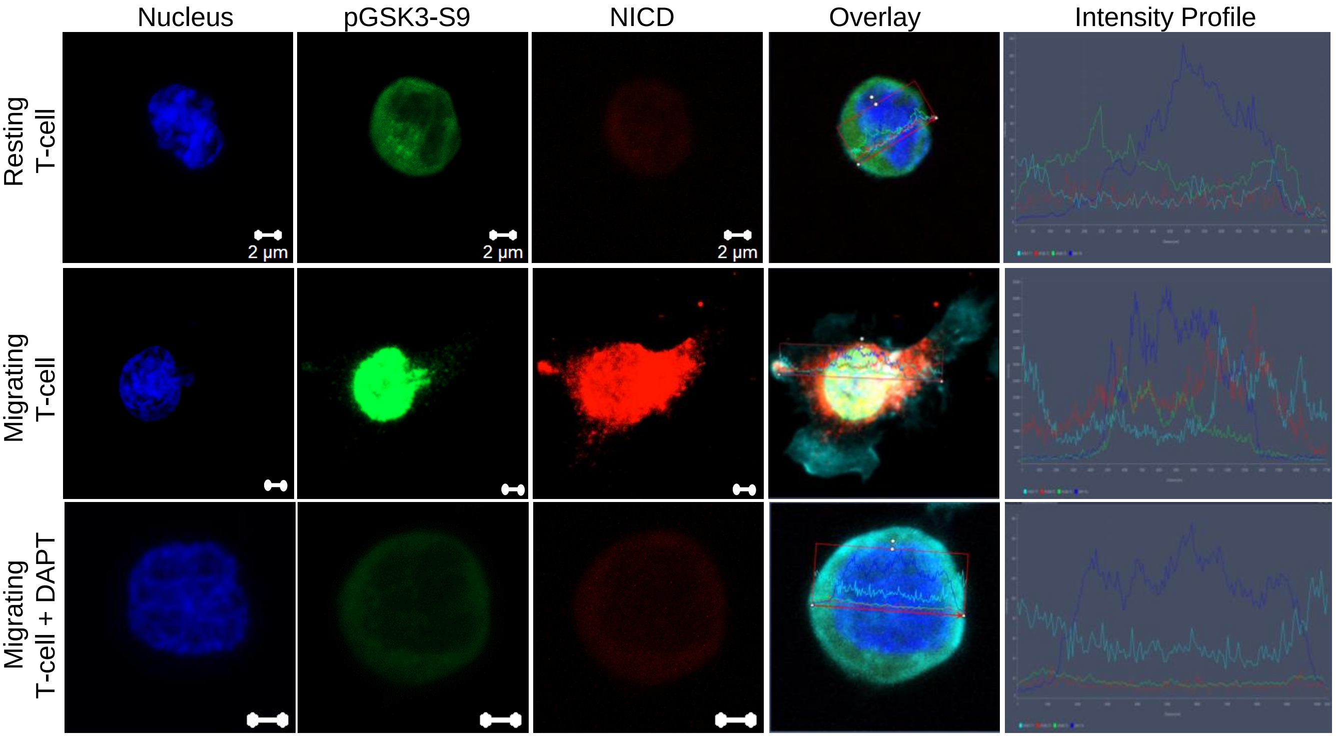

Supplementary Figure S4. GSK3β-Notch1 interactions in motile T-cells. Resting and LFA-1-stimulated HuT78 T-cells were immunostained with anti-pGSK3β-S9/Alexa Fluor® 488 (*green*), anti-NICD/Alexa Fluor® 568 (*red*), phalloidin-Alexa Fluor® 647 (Actin, *cyan*) and Hoechst (Nucleus, *blue*) and then imaged by confocal laser scanning microscopy, array scan 63X oil objective. Intensity profiles of nucleus, pGSK3β-S9, NICD and actin are shown. Data represent at least three independent experiments. Scale bar = 2 µm.

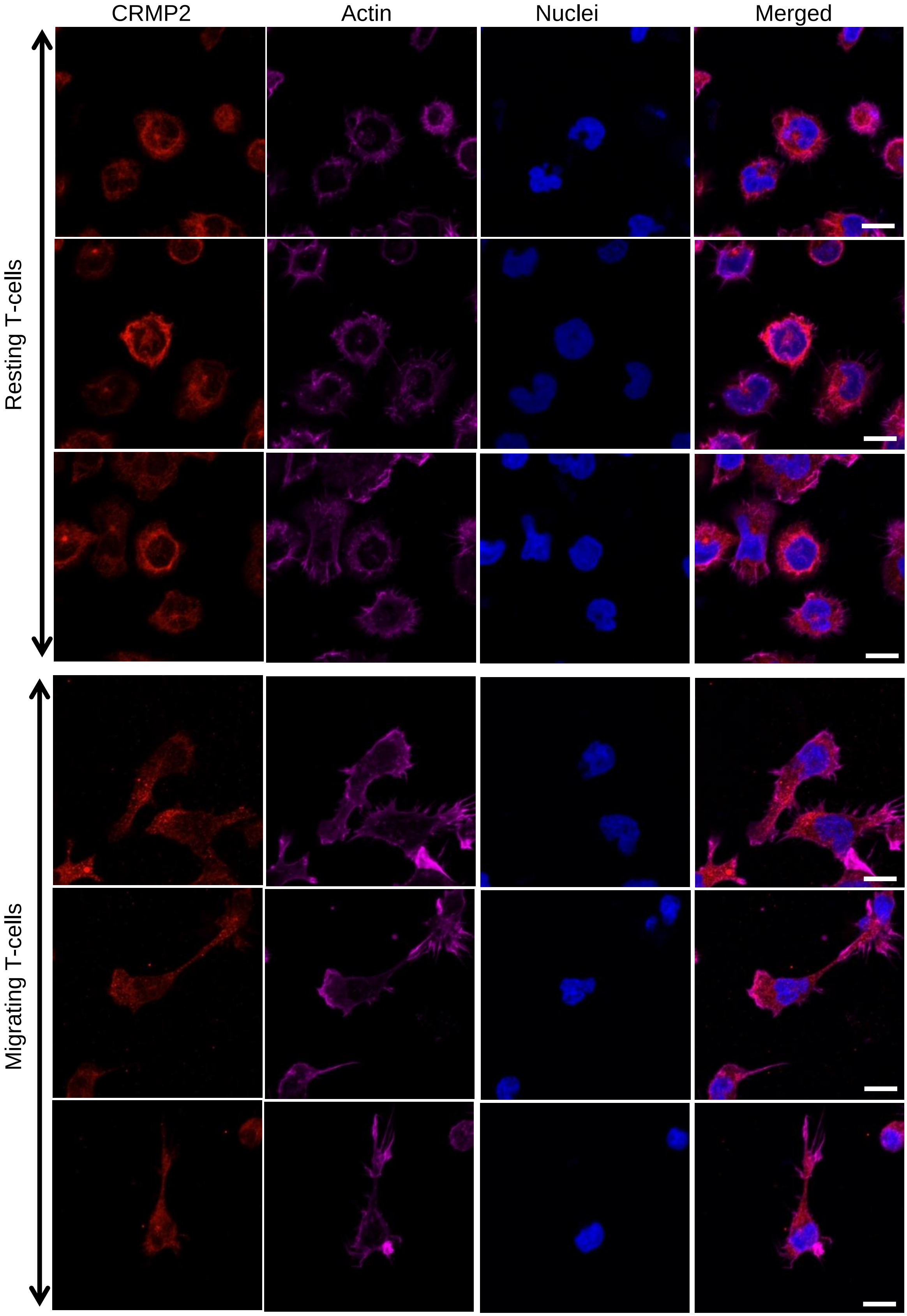

Supplementary Figure S5. Cellular distribution of CRMP2 in HuT78 T-cells. Resting and LFA-1-stimulated migrating HuT78 T-cells were immunostained with anti-CRMP2/Alexa Fluor® 568 (*red*), phalloidin-Alexa Fluor® 647 (actin, *pink*) and Hoechst (nucleus, *blue*). Cells were then imaged by confocal laser scanning microscopy, 63X oil objective. Scale bar = 5 μm. Three representative image sets from at least three independent experiments are presented.

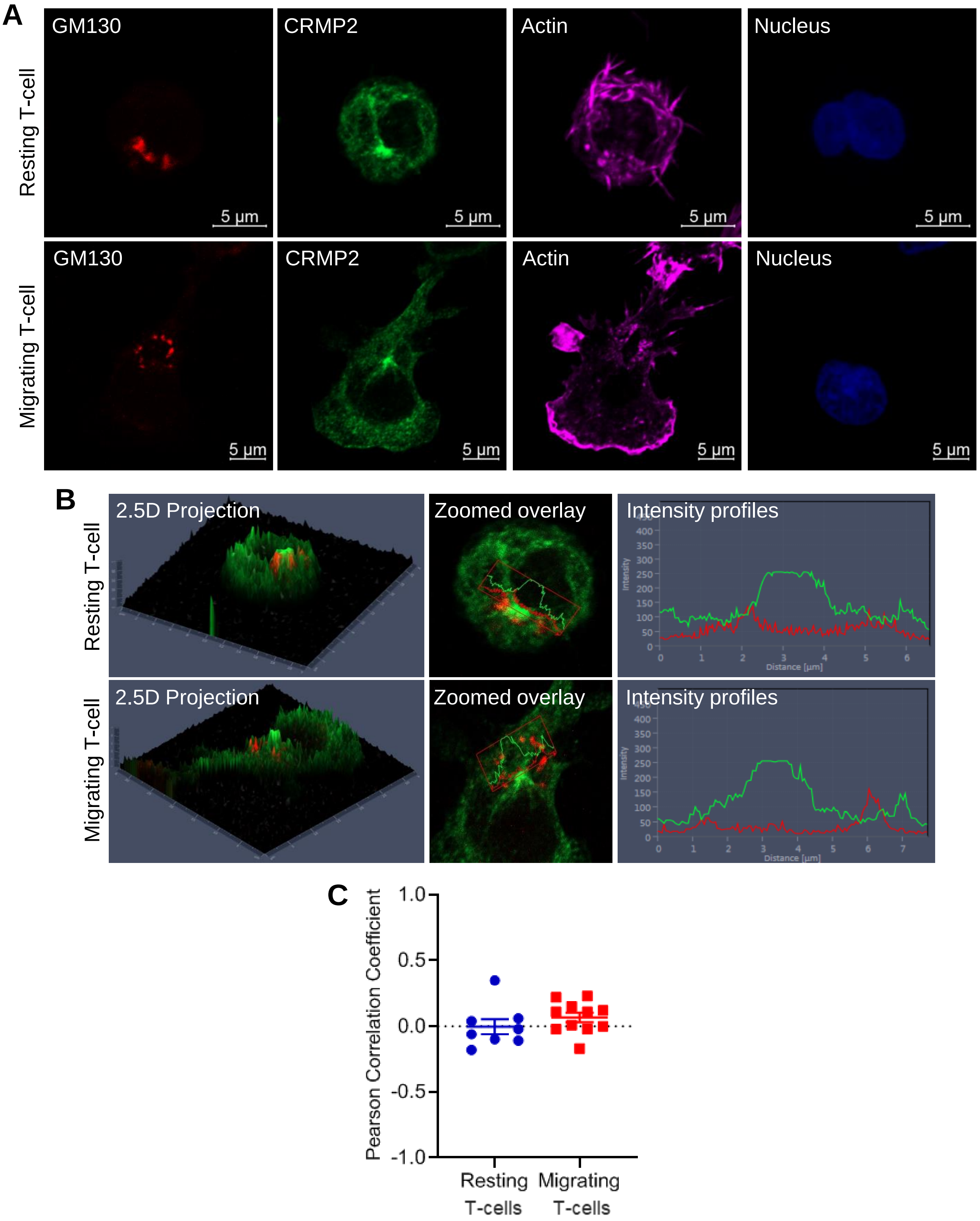

Supplementary Figure S6. Determination of CRMP2 localization to the Golgi in motile T-cells. (**A**) Resting and LFA-1-stimulated migrating HuT78 T-cells were immunostained with anti-GM130/Alexa Fluor® 568 (*red*), anti-CRMP2/Alexa Fluor® 488 (*green*), phalloidin-Alexa Fluor® 647 (actin, *pink*) and Hoechst (nucleus, *blue*). Cells were then imaged by confocal laser scanning microscopy, array scan 63X oil objective. (**B**) 2.5D projection, zoomed overlay images and intensity profiles of CRMP2 and GM130 are shown. (**C**) Colocalization of CRMP2 with GM130 was assessed by the Pearson Correlation Coefficient quantified using ZEN Black software (Carl Zeiss) and plotted using GraphPad Prism 8.1.0. Each dot represents a single T-cell, and the images were taken from at least three independent experiments; n=8 for resting T-cells and n=11 for migrating T-cells; error bar mean ± SEM.

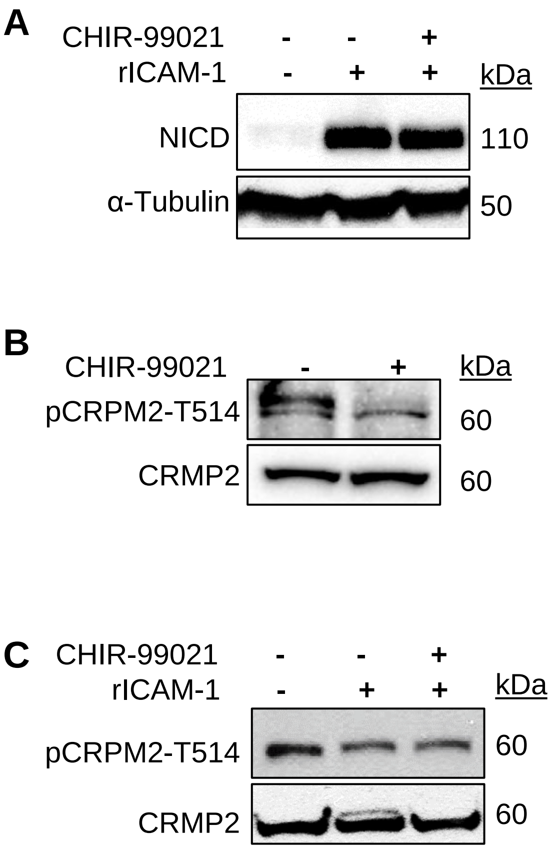

Supplementary Figure S7. Effect of CHIR-99021 on LFA-1-induced Notch1 cleavage and CRMP2 phosphorylation in motile T-cells. Human primary T-cells were pre-treated with CHIR-99021 for 2 h before stimulating them to migrate on rICAM-1-coated plate for 30 min. Untreated or unstimulated T-cells were used as controls. Cells were lysed and Western immunoblotted for cleaved Notch1 (NICD) (**A**) and phospho-CRMP2 (pCRMP2-T514) (**B, C**). Blots were re-probed with anti-α-tubulin or anti-CRMP2 as loading control. Data represent at least two independent experiments performed by using PBL T-cells derived from at least 2 different donors.
